# Supplementary material for: Resolving the full spectrum of human genome variation using Linked-Reads
Source: Genome Res. 2019 Apr;29(4):635–45. doi: 10.1101/gr.234443.118 (PMC6442396; doi:10.1101/gr.234443.118)
Supplement: Supplemental Material [file supp_gr.234443.118_Supplemental_longranger-2.2.zip › longranger-2.2.tar.bz2!/lr2.2-public/lib/stan/cmdstan/stan/doc/api/html/agrad_2fwd_2functions_2log__sum__exp_8hpp_source.html]

Stan: src/stan/agrad/fwd/functions/log\_sum\_exp.hpp Source File


|  |  |
| --- | --- |
|  | Stan  2.5.0  probability, sampling & optimization |


- Main Page
- Related Pages
- Namespaces
- Classes
- Files

- File List
- File Members

All Classes Namespaces Files Functions Variables Typedefs Enumerations Enumerator Friends Macros Pages

- src
- stan
- agrad
- fwd
- functions

log\_sum\_exp.hpp

Go to the documentation of this file.

1 #ifndef STAN\_\_AGRAD\_\_FWD\_\_FUNCTIONS\_\_LOG\_SUM\_EXP\_HPP

2 #define STAN\_\_AGRAD\_\_FWD\_\_FUNCTIONS\_\_LOG\_SUM\_EXP\_HPP

3

4 #include <stan/agrad/fwd/fvar.hpp>

5 #include <stan/meta/traits.hpp>

6 #include <stan/math/functions/log\_sum\_exp.hpp>

7

8 namespace stan {

9

10  namespace agrad {

11

12  template <typename T>

13  inline

14  fvar<T>

15  log\_sum\_exp(const fvar<T>& x1, const fvar<T>& x2) {

16  using stan::math::log\_sum\_exp;

17  using std::exp;

18  return fvar<T>(log\_sum\_exp(x1.val\_, x2.val\_),

19  x1.d\_ / (1 + exp(x2.val\_ - x1.val\_))

20  + x2.d\_ / (exp(x1.val\_ - x2.val\_) + 1));

21  }

22

23  template <typename T>

24  inline

25  fvar<T>

26  log\_sum\_exp(const double x1, const fvar<T>& x2) {

27  using stan::math::log\_sum\_exp;

28  using std::exp;

29  return fvar<T>(log\_sum\_exp(x1, x2.val\_),

30  x2.d\_ / (exp(x1 - x2.val\_) + 1));

31  }

32

33  template <typename T>

34  inline

35  fvar<T>

36  log\_sum\_exp(const fvar<T>& x1, const double x2) {

37  using stan::math::log\_sum\_exp;

38  using std::exp;

39  return fvar<T>(log\_sum\_exp(x1.val\_, x2),

40  x1.d\_ / (1 + exp(x2 - x1.val\_)));

41  }

42

43  template <typename T>

44  fvar<T>

45  log\_sum\_exp(const std::vector<fvar<T> >& v) {

46  using stan::math::log\_sum\_exp;

47  using std::exp;

48  std::vector<T> vals(v.size());

49  for (size\_t i = 0; i < v.size(); ++i)

50  vals[i] = v[i].val\_;

51  T deriv(0.0);

52  T denominator(0.0);

53  for (size\_t i = 0; i < v.size(); ++i) {

54  T exp\_vi = exp(vals[i]);

55  denominator += exp\_vi;

56  deriv += v[i].d\_ \* exp\_vi;

57  }

58  return fvar<T>(log\_sum\_exp(vals), deriv / denominator);

59  }

60

61  }

62 }

63 #endif

log\_sum\_exp.hpp

stan::agrad::log\_sum\_exp

fvar< T > log\_sum\_exp(const fvar< T > &x1, const fvar< T > &x2)

**Definition:** log\_sum\_exp.hpp:15

stan::math::log\_sum\_exp

boost::math::tools::promote\_args< T1, T2 >::type log\_sum\_exp(const T2 &a, const T1 &b)

Calculates the log sum of exponetials without overflow.

**Definition:** log\_sum\_exp.hpp:49

stan::agrad::fvar

**Definition:** fvar.hpp:13

traits.hpp

fvar.hpp

stan::agrad::fvar::d\_

T d\_

**Definition:** fvar.hpp:16

stan::agrad::exp

fvar< T > exp(const fvar< T > &x)

**Definition:** exp.hpp:16

stan::agrad::fvar::val\_

T val\_

**Definition:** fvar.hpp:15


---

[ Stan Home Page ]

*© 2011–2014,
Stan Development Team.*
